# Supplementary figures and images for: Analyzing game statistics and career trajectories of female elite junior tennis players: A machine learning approach
Source: PLoS One. 2023 Nov 30;18(11):e0295075. doi: 10.1371/journal.pone.0295075 (PMC10688900; doi:10.1371/journal.pone.0295075)

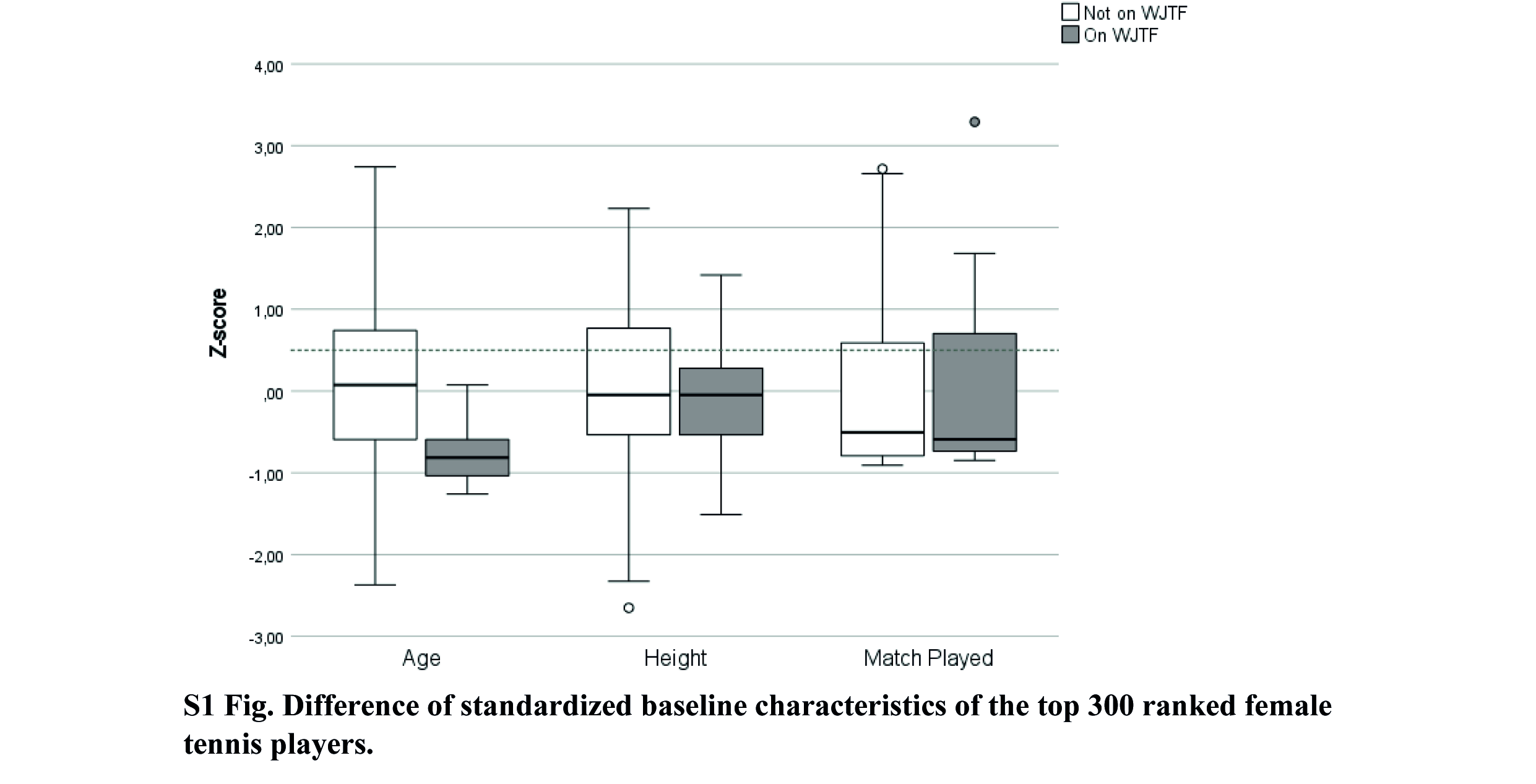

Supplement: S1 Fig — (TIF) [file pone.0295075.s001.tif]

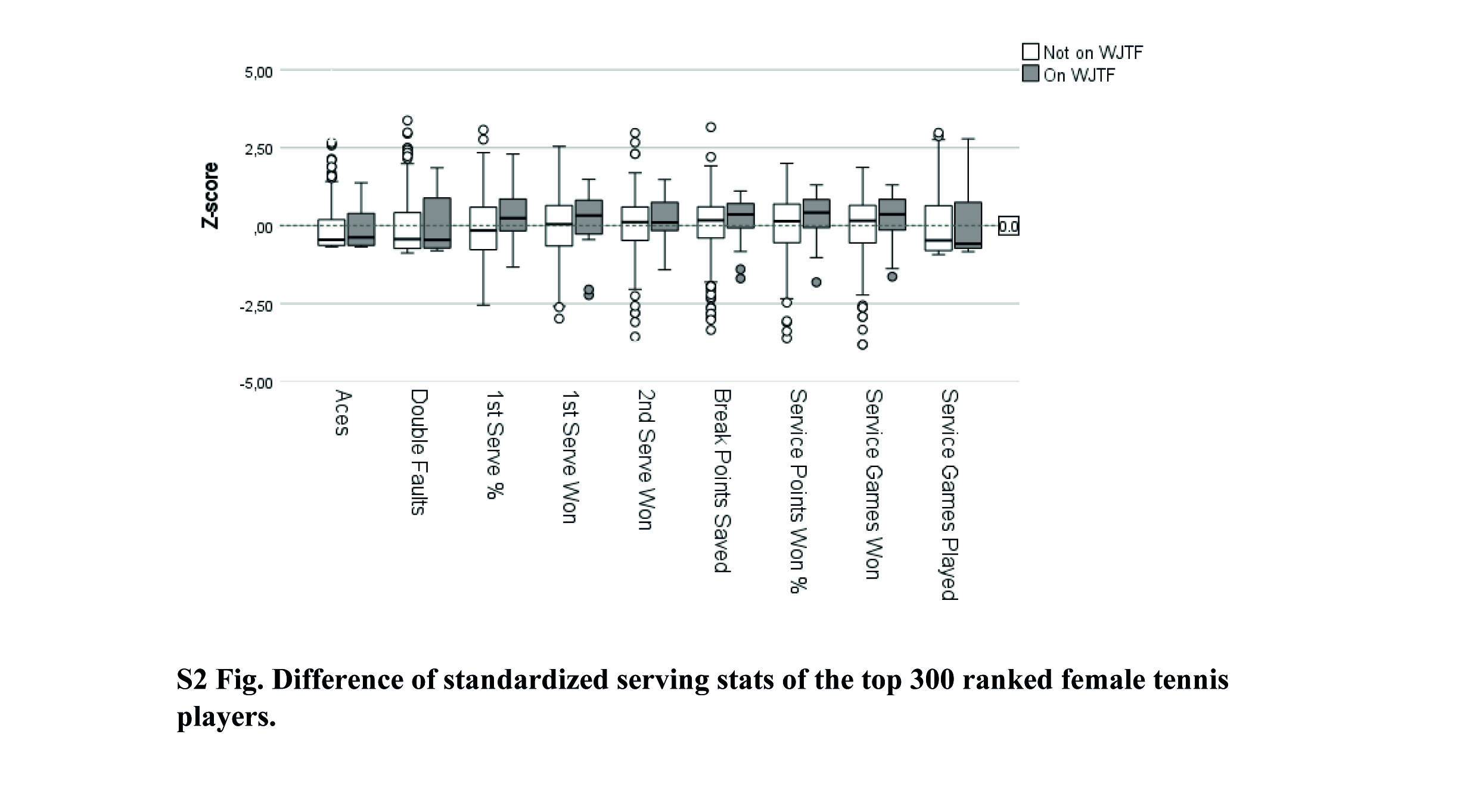

Supplement: S2 Fig — (TIF) [file pone.0295075.s002.tif]

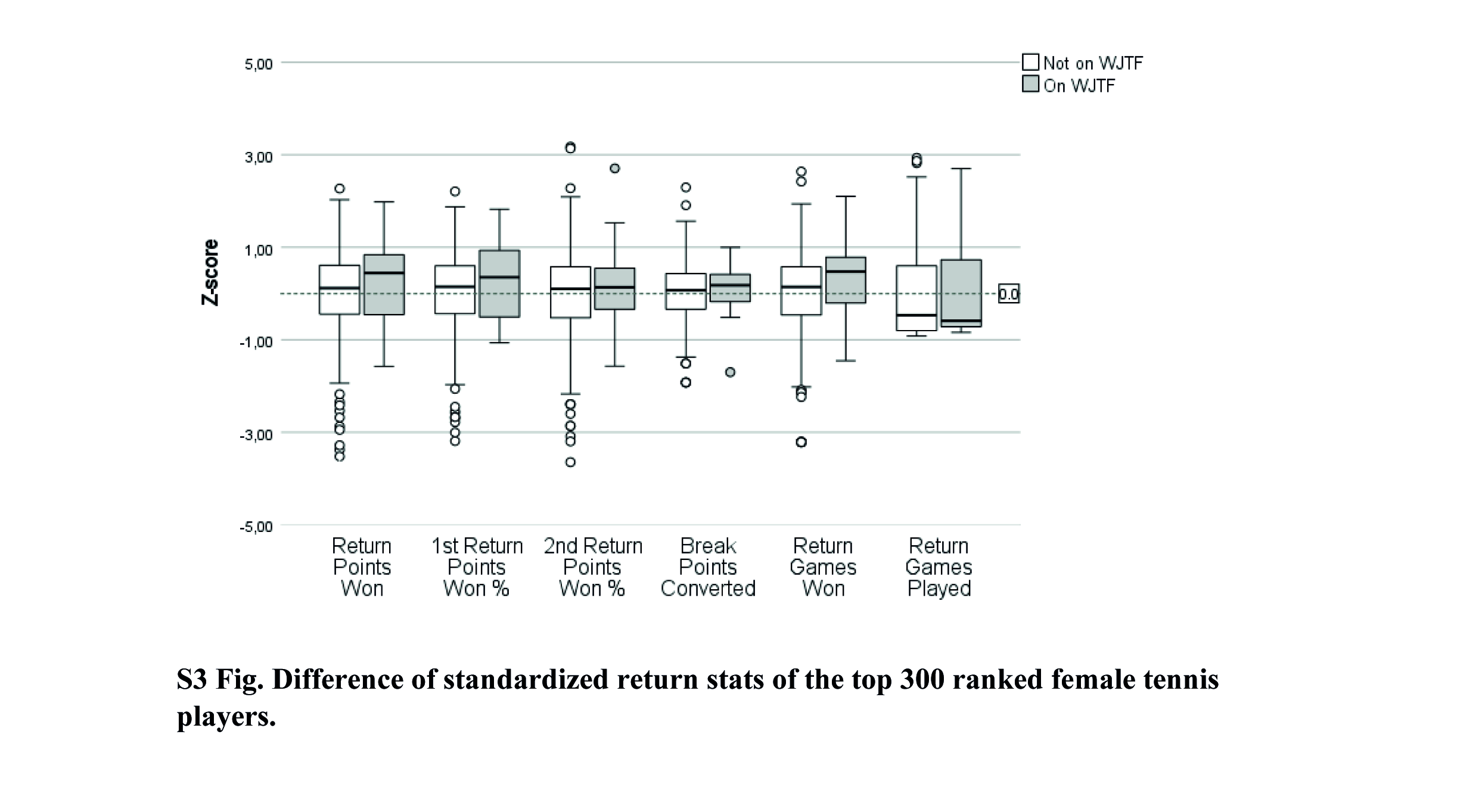

Supplement: S3 Fig — (TIF) [file pone.0295075.s003.tif]

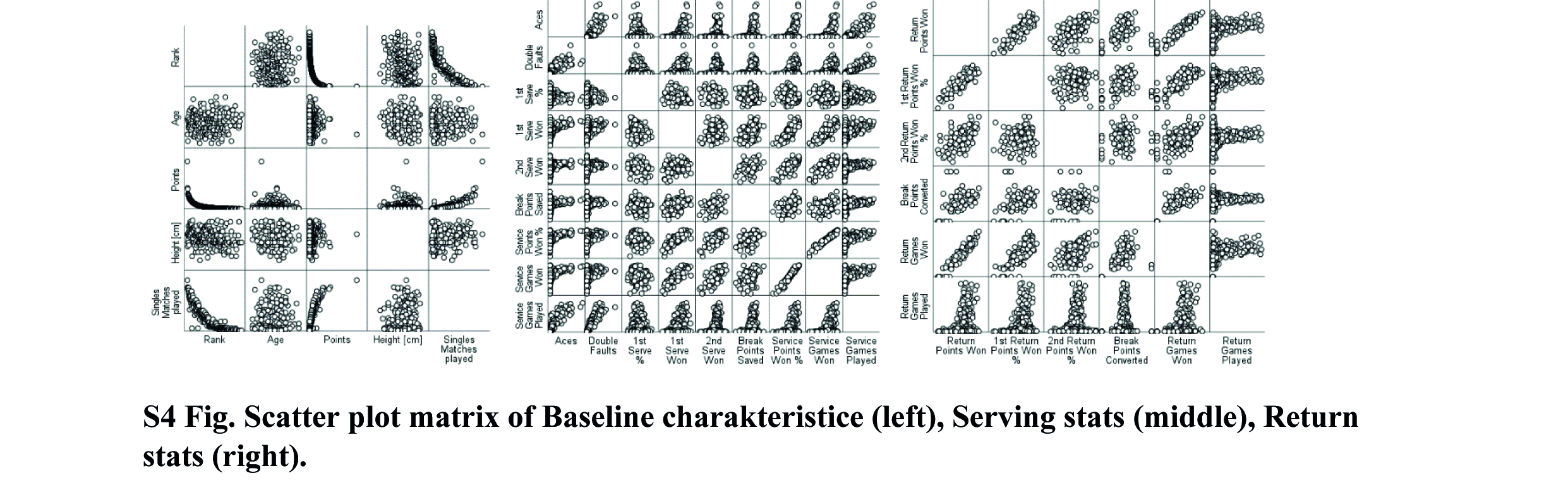

Supplement: S4 Fig — (TIF) [file pone.0295075.s004.tif]
