# Supplementary material for: Analyzing game statistics and career trajectories of female elite junior tennis players: A machine learning approach
Source: PLoS One. 2023 Nov 30;18(11):e0295075. doi: 10.1371/journal.pone.0295075 (PMC10688900; doi:10.1371/journal.pone.0295075)
Supplement: S1 Table — (DOCX) [file pone.0295075.s005.docx]

**S1 Table. An overview of the input and output type and use in classification models.**

| **Variable** | **Scale** | **The Classification Models** | | | |
| --- | --- | --- | --- | --- | --- |
|  |  | **1** | **2** | **3** | **4** |
| Nomination | Ordinal | Output | Input | Input | Input |
| Tournament year | Ordinal | Input | Input | Input | Input |
| Country | Nominal | Input | Input | Input | Input |
| Continent | Nominal | Input | Input | Input | Input |
| Birth year | Ordinal | Input | Input | Input | Input |
| Tournament Final Rank | Ordinal | Input | Output | Input | Input |
| Ranking at WTA | Dichotomic | - | - | Output | - |
| Ranking at WTA (cat.) | Ordinal | - | - | - | Output |
