# Supplementary material for: Analyzing game statistics and career trajectories of female elite junior tennis players: A machine learning approach
Source: PLoS One. 2023 Nov 30;18(11):e0295075. doi: 10.1371/journal.pone.0295075 (PMC10688900; doi:10.1371/journal.pone.0295075)
Supplement: S2 Table — (DOCX) [file pone.0295075.s006.docx]

**S2 Table. Overview of used variables for the neural network.**

| **Variable** | **Type** | **Scale** | **The Neural network Models** | | | | | |
| --- | --- | --- | --- | --- | --- | --- | --- | --- |
|  |  |  | **All** | **Baseline characteristic** | **Serving stat** | **Serving stats %** | **Return stat** | **Return stat %** |
| No. inputs/output |  |  | 18/1 | 3/1 | 9/1 | 6/1 | 6/1 | 5/1 |
| Age | Input | Continuous | ● | ● |  |  |  |  |
| Height | Input | Continuous | ● | ● |  |  |  |  |
| Singles Matches played | Input | Count | ● | ● |  |  |  |  |
| Aces | Input | Count | ● |  | ● |  |  |  |
| Double Faults | Input | Count | ● |  | ● |  |  |  |
| 1^st^ Serve % | Input | Percentage | ● |  | ● | ● |  |  |
| 1st Serve Won | Input | Percentage | ● |  | ● | ● |  |  |
| 2nd Serve Won | Input | Percentage | ● |  | ● | ● |  |  |
| Break Points Saved | Input | Percentage | ● |  | ● | ● |  |  |
| Service Points Won % | Input | Percentage | ● |  | ● | ● |  |  |
| Service Games Won | Input | Percentage | ● |  | ● | ● |  |  |
| Service Games Played | Input | Count | ● |  | ● |  | ● | ● |
| Return Points Won | Input | Percentage | ● |  |  |  | ● | ● |
| 1^st^ Return Points Won % | Input | Percentage | ● |  |  |  | ● | ● |
| 2^nd^ Return Points Won % | Input | Percentage | ● |  |  |  | ● | ● |
| Break Points Converted | Input | Percentage | ● |  |  |  | ● | ● |
| Return Games Won | Input | Percentage | ● |  |  |  | ● | ● |
| Return Games Played | Input | Count | ● |  |  |  | ● |  |
| Rank | Output | Ordinal | ● | ● | ● | ● | ● | ● |
