# Supplementary material for: Analyzing game statistics and career trajectories of female elite junior tennis players: A machine learning approach
Source: PLoS One. 2023 Nov 30;18(11):e0295075. doi: 10.1371/journal.pone.0295075 (PMC10688900; doi:10.1371/journal.pone.0295075)
Supplement: Attachment — Submitted filename: Response to Reviewers.docx [file pone.0295075.s008.docx]

**Journal Requirements:**

review your reference list to ensure that it is complete and correct

- We have checked the references list, and it is complete and correct.

**Additional Editor Comments:**

Technically, the paper is well-executed. The introduction is well-structured, and the purpose is clear.

- Thank you for your positive feedback on our work.

I would ask the authors to provide a more detailed explanation for their focus solely on women and the exclusion of men (recognizing the general lack of women-focused studies, this should be further justified).

- In the manuscript, they expanded and added the following justification for why the study focuses solely on a female sample:
  - „This study focused solely on junior and professional senior female tennis players. It did not include a male sample due to significant disparities in performance between sexes at the junior and professional senior levels [1,12,56]. Even if it were possible (probably with sufficient accuracy based on the results from the literature) to predict gender and age level, the practical applicability of this model would be minimal. Since the error rate of this model would negatively affect the results of the current study, we limited our focus to female tennis players. This decision was also influenced by the absence of similar studies targeting girls/women. Analyzing and interpreting the trajectories and performances of men and women would have significantly lengthened the manuscript. Additionally, differences in variables at the official ATP and WTA websites would present problems for data comparison. “
- Thank you for this comment, and we hope it will serve as sufficient justification.

The methods are clear and well-described.

- Thank you for the positive and appreciated feedback.

However, the decision to incorporate **results and discussion** within the same paragraphs is stylistically questionable. It might be more effective to present the results first and then discuss them separately.

- Based on this recommendation, we divided this chapter into two separate chapters. We deleted the relevant text from the results and reformatted it into the discussion chapter, to which we added new citations. Specifically, there were 10 citations added.

Additionally, there are some typographical errors in the text, suggesting a thorough proofreading (preferably by a native speaker) is needed.

- We have ensured proofreading and corrected typos in the text.
